# Supplementary material for: Oxidative Stress Causes Vacuolar Fragmentation in the Human Fungal Pathogen Cryptococcus neoformans
Source: J Fungi (Basel). 2021 Jun 29;7(7):523. doi: 10.3390/jof7070523 (PMC8305764; doi:10.3390/jof7070523)
Supplement: Supplementary file 1 [file jof-07-00523-s001.zip › jof-1246527-supplementary.pdf]

Table S1. Primers used in this study

Primers	Sequence (5′to 3′)
Sod1-GFP_F	GCTCTCACCGTATCAAGCTTTGCTGG
Sod1-GFP_R	GAATCTGGATCCGGTAGAAATACCGCTGCGAATAC
Sod2-GFP_F	GGATCTAAGCTTTGAGCTAAATCCATGTGAGCTTCCCTG
Sod2-GFP_R	GAATCTGGATCCCTGAGCAGCCTTGAGACGACTCTC
FAB1_KO1	GCGGCTGTTAACTGTTGATGGTTCTAAGG
FAB1_KO2	AATTCTGCAGATATCCATCACACTGGCGGCCAGAGCTACTGTGGGGATCAAGTGAA
FAB1_KO3	AATTCCAGCACACTGGCGGCCGTTACTAGTAGCCTGGGATTTGAAGCACATTTAGC
FAB1_KO4	GAGTGCCTTGCAGTGAGTAAGATATC
FAB1_KO5	GGTGATTTATGTACTGCTGAGCGCAG
FAB1_KO6	GATCTTCTCTTGAGAAGGGCCAAGAC

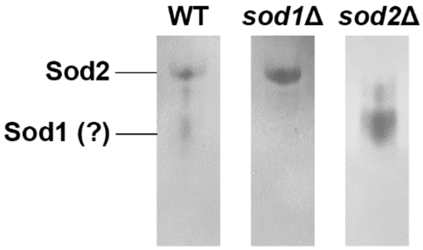

Figure S1. Superoxide dismutase activity assay. The upper band, which represents the Sod2 activity was disappeared in the *sod2* mutant. The lower faint band represents the Sod1 activity.
